# Agreement and repeatability of ocular surface function using the S390L Firefly WDR slitlamp compared with Keratograph 5M

**DOI:** 10.1038/s41598-025-18904-z

**Published:** 2025-10-07

**Authors:** Linlin Su, Tingting Yu, Jiayan Chen, Fan Yang, Qiuyue Zhang, Ling Xu, Wei He, Guanghao Qin, Hongda Zhang

**Affiliations:** 1 Department of Clinical Research, He Eye Specialist Hospital, Shenyang, 110034 China; 2 Department of Ophthalmology, Dalian Medical University, Dalian, China

**Keywords:** Dry eye, Tear film, S390L Firefly WDR slitlamp, Keratograph 5M, Diseases, Health care, Medical research

## Abstract

To evaluate the agreement and repeatability of the S390L Firefly WDR slitlamp (S390L WDR+D130, MediWorks, Shanghai, China)   in tear film-related parameters measurement compared with Keratograph 5M(K5M) (Oculus Optikgeräte GmbH, Wetzlar, Germany). This prospective study assessed tear film parameters, including first non-invasive tear break-up time (NIBUTf), average non-invasive tear break-up time (NIBUTav), and tear meniscus height (TMH) using the S390L Firefly WDR slitlamp and K5M. Bland-Altman (BA) plots and the correlation coefficient r were used to assess the agreement of tear film parameters of two ocular surface analyzers. Intraclass correlation coefficient (ICC) was used to evaluate the repeatability of the S390L Firefly WDR slitlamp and K5M. Forty-four subjects from Shenyang He Eye Specialist Hospital were recruited in this study. There were no significant differences in the paired comparisons of NIBUTf, NIBUTav, and TMH (*p* > 0.05). The BA plots analysis showed a good agreement between the two devices for the NIBUTf (95% LoA, − 2.7 to 2.6), NIBUTav (95% LoA, − 4.5 to 3.6) and TMH (95% LoA, − 0.05 to 0.04). ICC of NIBUTf, NIBUTav, and TMH between the two measurements using S390L Firefly WDR slitlamp was 0.89, 0.84, and 0.98, respectively. The tear film-related parameters measurement of S390L Firefly WDR slitlamp had good repeatability and acceptable agreement with K5M. This study suggested a novel technique S390L Firefly WDR slitlampcan be considered an alternative to K5M in clinical settings, with an acceptable level of intrasession repeatability for clinical evaluation of tear film parameters.

## Introduction

Dry eye disease (DED) is a pathological condition of the ocular surface characterized by an instability of the tear film. This instability is primarily caused by a decrease in tear secretion (hyposecretion) or an increase in tear evaporation^1,2^. DED is a prevalent and persistent ocular disorder that significantly impacts individuals’ quality of life. It is estimated that DED affects approximately 14–35% of adults^3,4^.

The tear film, ranging in thickness from 2.0 to 5.5 μm, is highly advanced in its function and composition and plays a crucial role in maintaining the physiology of the ocular surface^5–7^. The tear film’s three-layer structure ensures stability through various mechanisms. The lipid layer prevents evaporation, the aqueous layer contributes to volume and lubrication, and the mucin layer reduces the hydrophobicity of the corneal epithelium^8^. The evaluation of tear film stability is crucial for diagnosing DED, particularly for assessing its stability^9^. Accurate tear film evaluation is crucial for diagnosing DED and ensuring proper fitting of contact lenses^10^.

The assessment of tear film-related parameters can be measured using the Schirmer test, tear film break-up time, and tear meniscus height (TMH). There are two primary measurement methods: invasive and non-invasive. Invasive methods may reduce patient comfort, causing discomfort, mild pain, or irritation during the evaluations. In recent years, the diagnosis of DED has increasingly incorporated multifunctional non-invasive instruments capable of performing multiple assessments within a single platform. These devices offer objective and non-invasive evaluations of the ocular surface, enabling more comprehensive and efficient clinical workflows^11,12^. Of particular importance is their ability to assess tear film dynamics under natural physiological conditions, thereby reducing potential artifacts caused by external interventions that may otherwise influence or bias the results^11^. Multiple video topographers, such as the Oculus Keratograph 5M (K5M) (Oculus Optikgeräte GmbH, Wetzlar, Germany), Medmont E300(Medmont Pty., Ltd., Melbourne, Australia), and the S390L Firefly WDR slitlamp (S390L WDR+D130, MediWorks, Shanghai, China) , utilize the reflection of Placido’s disk mires to evaluate the tear film’s quality in a non-invasive manner. Each device utilizes unique algorithms to determine the non-invasive tear film break-up time (NIBUT)^11,12^. TMH serves as a parameter for indirectly assessing tear volume, which can facilitate the sub-classification of DED^13^.

The K5M is among the most frequently utilized instruments for evaluating the ocular surface layer and a corneal topographer that utilizes automated real-time videokeratoscopy analysis to measure NIBUT and TMH^14^. The illuminator of the K5M incorporates 200 red light-emitting diodes that emit light at a wavelength of 880 nm^13^. This specific wavelength prevents thermal variations in the lacrimal film^1^.

The S390L Firefly WDR slitlamp assesses parameters such as tear film stability and evaporation rate to generate comprehensive reports on ocular surface health^11^. It utilizes a Placido ring projection system with visible light for NIBUT examinations, covering a broad scope of up to an 8 mm corneal diameter. The AI identification system visualizes the tear meniscus area and automatically measures tear height, objectively evaluating tear secretion amount and continuity. This study aimed to evaluate the agreement and repeatability between the S390L Firefly WDR slitlamp and the K5M measurement system in assessing tear film-related parameters.

## Methods

### Ethics approval

This prospective study, conducted between December 2023 and May 2024, received ethical approval from the Human Subject Ethics Subcommittee of Shenyang He Eye Specialist Hospital. (IRB (2023) K004.01) Assent and informed consent were obtained from subjects after fully explaining and disclosing the study’s objectives before commencing the study. The principles of the Declaration of Helsinki were followed throughout all procedures.

### Subjects

Forty-four subjects (18 males and 26 females) were enrolled in the study voluntarily, with unremarkable ocular health. The inclusion criteria for this study were individuals aged 18 and above. The exclusion criteria included a history of eye surgery, corneal ulcer, corneal scarring, keratoconus, ocular trauma, glaucoma, acute conjunctivitis, and dacryocystitis. The subjects were instructed to abstain from wearing contact lenses and using any form of eye drops on the day of their visit.

### Examination procedures

**Fig. 1:**
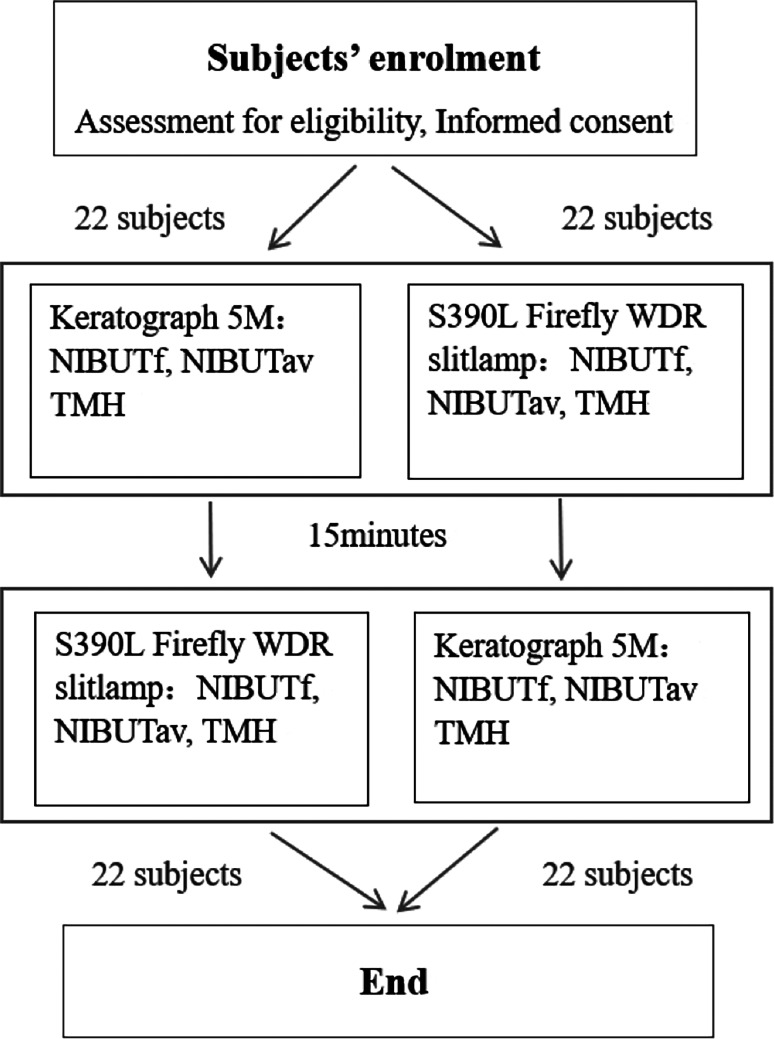
Study design. NIBUTf, first non-invasive tear break-up time; NIBUTav, average non-invasive tear break-up time; TMH, tear meniscus height.

The 44 subjects were randomly assigned into two groups, with 22 individuals initially tested by K5M and the remaining individuals initially tested by the S390L Firefly WDR slitlamp. (Fig. 1) There was no stratification by gender or age. All tests were conducted between 8:00 a.m. and 12:00 noon to consider potential temporal variations and maintain consistency in tear film parameters.

Subjects were advised not to intentionally withhold blinking for an extended period to avoid reflex tearing. The same experienced doctor performed the examinations, and participants were instructed not to use any additional dry eye medications or eye drops, including preservative-free artificial tears, throughout the study. Measurements were obtained from both eyes; however, the analysis was performed exclusively on the measurements from the right eye.

### Statistical analysis

Statistical analysis was conducted using IBM SPSS Statistics version 26.0 for Windows (IBM Corp, Armonk, New York, USA). The results are presented as the mean ± standard deviation (SD) and median. Analysis was conducted on the right-eye data from all participants. Paired sample t-tests were used for datasets that showed a normal distribution. To evaluate the agreement between the two ocular surface analyzers for tear film parameters, Bland-Altman (BA) plots were generated. In addition, the correlation coefficient (r) was computed to assess the correlation between the two measurement methods. The repeatability of each parameter was evaluated using the Intraclass correlation coefficient (ICC). The 95% confidence interval was calculated. The reliability of the measurements was classified based on the ICC values: ICC values less than 0.50 were classified as poor, values ranging from 0.50 to 0.75 were classified as moderate, values ranging from 0.75 to 0.90 were classified as good, and values above 0.90 were rated as acceptable. A p-value less than 0.05 was considered statistically significant. A two-sided matched pairs t-test was used to assess the difference between the means of two repeated measurements. G*Power 3.1.9.2 software was used to calculate the sample size of the paired T-test. Assuming that the effect size of the two devices was 0.5 and the alpha error was 5%, at least 38 people were needed to achieve the effect of 85% power.

## Results

The final analysis was based on data from the right eyes of 44 participants (18 males and 26 females), with ages ranging from 24 to 48 years (mean ± SD: 36 ± 12 years).

### Agreement assessment

Table 1 summarizes the agreement between the two devices in measuring the tear film. The paired comparisons of the first NIBUT (NIBUTf), average NIBUT (NIBUTav), and TMH showed no significant differences (*p* > 0.05). The BA plot analysis revealed a strong agreement between the two instruments for the NIBUTf (95% limits of agreement (LoA): −2.7 to 2.6), NIBUTav (95% LoA: −4.5 to 3.6), and TMH (95% LoA: −0.05 to 0.04), with narrow limits of agreement. The ICC for NIBUTf between the two devices was 0.96 (95% confidence interval (CI): 0.93 to 0.98, *p* < 0.001). The ICC for NIBUTav between the two devices was 0.92 (95% CI: 0.85 to 0.95, *p* < 0.001). The ICC for TMH between the two devices was 0.97 (95% CI: 0.94 to 0.98, *p* < 0.001). There is a strong correlation between the K5M and S390L Firefly WDR slitlamp in measuring the ocular surface parameters, including NIBUTf, NIBUTav, and TMH. The correlation coefficients r for NIBUTf, NIBUTav, and TMH were 0.97, 0.92, and 0.93, respectively (*p* < 0.001).

**Table 1:** The agreement of ocular surface parameter measurements between two groups of comprehensive ocular surface analyzers.

	Keratograph 5M		S390L Firefly WDR slitlamp		*p*	*r*
	Mean (SD)	M (P25, P75)	Mean (SD)	M (P25, P75)		
NIBUTf (s)	9.50 (5.25)	7.99(5.49,13.61)	9.57(4.70)	8.67(6.17,12.00)	0.74	0.97^*^
NIBUTav (s)	10.00(5.05)	8.96(5.95,13.69)	10.44(5.23)	9.07(6.21,13.48)	0.16	0.92^*^
TMH (mm)	0.20(0.06)	0.18(0.15,0.25)	0.20(0.06)	0.19(0.15,0.25)	0.08	0.93^*^

NIBUTf, first non-invasive tear break-up time; NIBUTav, average non-invasive tear break-up time; TMH, tear meniscus height; mm, millimeter; s, second; CI, confidence interval; SD, standard deviation; ICC, the intraclass correlation coefficient; *: *p* < 0.001; M (P25, P75): Median with interquartile range.

**Fig. 2:**
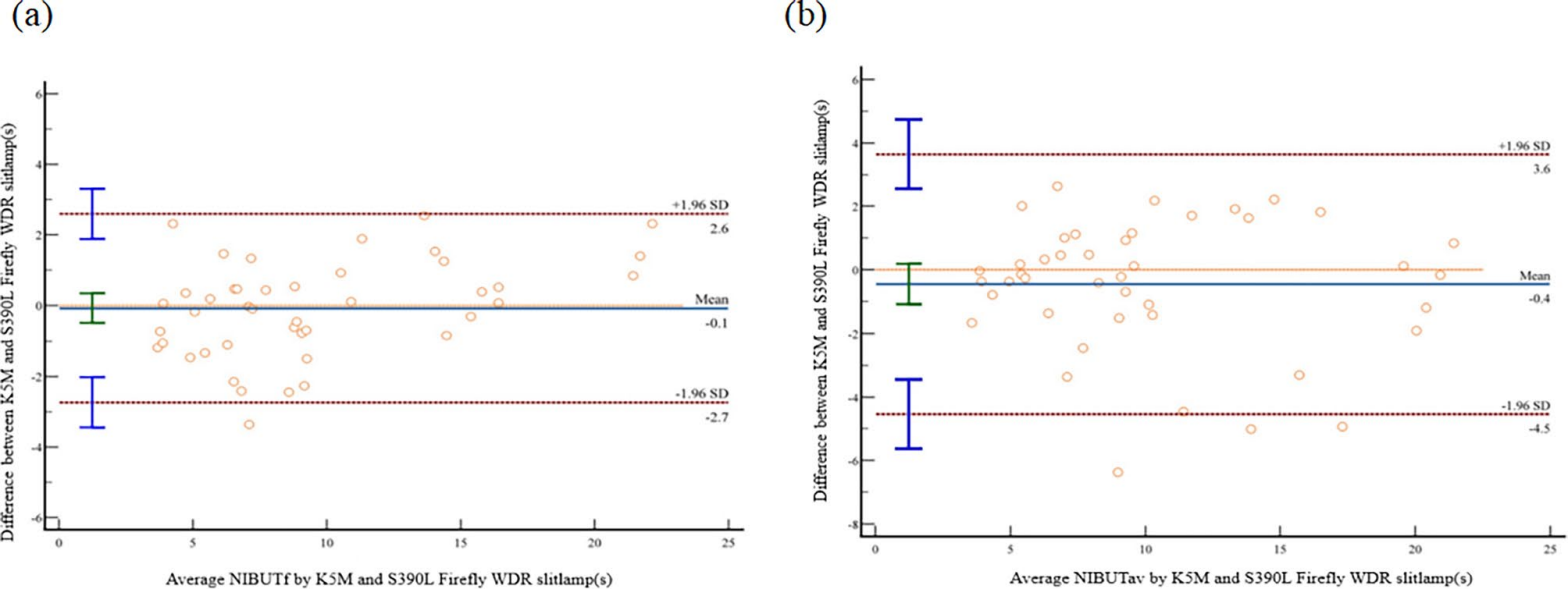
BA plot assessing the agreement between the K5M and S390L Firefly WDR slitlamp for measuring Average NIBUTf (**a**) and Average NIBUTav (**b**). The solid blue line indicates the mean difference. The dashed red lines represent the 95% limits of agreement (mean difference ± 1.96 SD). Individual paired measurements are plotted as orange circles. The vertical bars on the left side depict the SD of the differences, calculated at three distinct average value intervals.

**Fig. 3:**
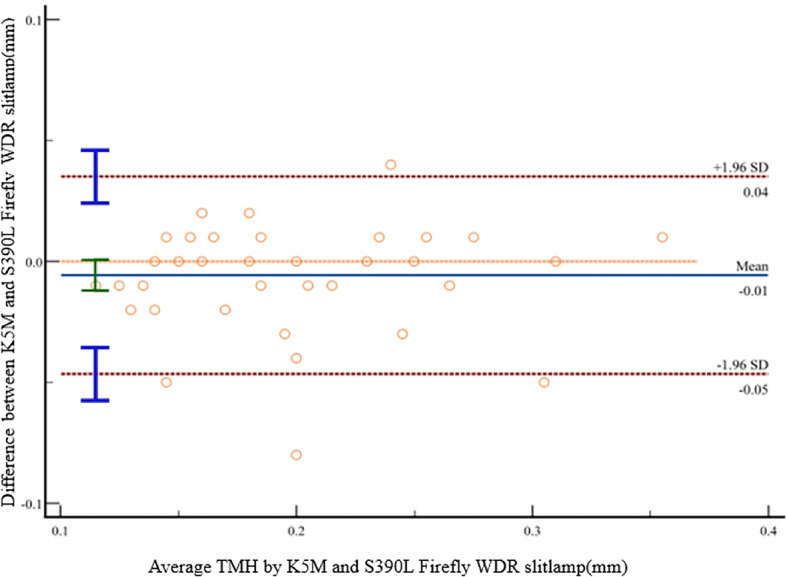
BA plot assessing the agreement between the K5M and S390L Firefly WDR slitlamp for measuring TMH. The solid blue line indicates the mean difference. The dashed red lines represent the 95% limits of agreement (mean difference ± 1.96 SD). Individual paired measurements are plotted as orange circles. The vertical bars on the left side depict the SD of the differences, calculated at three distinct average value intervals.

Non-parametric BA plots were used to depict the relationship between the differences in measurements from the two instruments and their average values for Fig. 2(a): NIBUTf, Fig. 2(b): NIBUTav, and TMH (Fig. 3). The upper and lower 95% limits of agreement are represented by the bilateral red dashed lines.

### Repeatability assessment

Table 2 demonstrates the within-test repeatability of tear film measurements. The ICC values of NIBUTf, NIBUTav, and TMH by the S390L Firefly WDR slitlamp measurements were 0.89, 0.84, and 0.98, respectively.

**Table 2:** Repeatability of ocular surface parameter measurements by two groups of comprehensive ocular surface analyzers.

		ICC	95%CI		p
			Lower	Upper	
NIBUTf	Keratograph 5M	0.92	0.86	0.96	0.001
	S390L Firefly WDR slitlamp	0.89	0.81	0.94	0.001
NIBUTav	Keratograph 5M	0.85	0.75	0.92	0.001
	S390L Firefly WDR slitlamp	0.84	0.90	0.96	0.001
TMH	Keratograph 5M	0.98	0.96	0.99	0.001
	S390L Firefly WDR slitlamp	0.98	0.96	0.99	0.001

NIBUTf, first non-invasive tear break-up time; NIBUTav, average non-invasive tear break-up time; TMH, tear meniscus height; ICC, the intraclass correlation coefficient; 95%CI, 95% confidence intervals.

## Discussion

This study assessed the agreement and repeatability of the S390L Firefly WDR slitlamp in measuring tear film-related parameters, compared to the K5M. The findings indicated that the tear film-related parameters measurement performed by S390L Firefly WDR slitlamp exhibited good repeatability and high agreement with the K5M. Although the 95% LoA for NIBUTf (− 2.7 to + 2.6 s) and NIBUTav (− 4.5 to + 3.6 s) may appear relatively wide, they are notably narrower than those reported in previous studies comparing different instruments for NIBUT measurement. For instance, a study evaluating the agreement between SBM Sistemi IDRA (IDRA) and K5M found substantially wider LoA, with ranges of − 8.13 to + 14.19 s for NIBUTf and − 7.89 to + 10.32 s for NIBUTav^12^. Given the known variability of NIBUT measurements due to physiological fluctuations and environmental conditions, the LoA observed in our study falls within a range that can be considered clinically acceptable and sufficiently precise for practical use. A study by Antonio et al.^11^ resulted that the S390L Firefly WDR slitlamp has moderate intraobserver repeatability for NIBUTf and NIBUTav, which suggestted that NIBUTf and NIBUTav are tests with high variability. The TMH values also show satisfactory intraobserver repeatability, which aligns with our findings. In contrast to our study, Ryan Lee et al.^16^ reported a weak concordance in NIBUT readings between the Tomey RT-7000 (from Tomey Corporation, Japan) and the K5M device. Based on the content of the article, an ICC of 0.187 was found between the RT-7000 and K5M for average measures, with a 95%CI of −0.097 to 0.406. This difference in findings could be explained by using different algorithms employed for measuring NIBUT. A weak level of agreement was observed between the Tomey RT-7000 and the K5M modalities. The RT-7000^16^ evaluates the entire tear film’s overall reflectivity by analyzing its reflective brightness. It captures one image per second, distributed across 15 additional rings. In contrast, the K5M employs infrared waves to evaluate tear film integrity, capturing images faster at 32 frames per second. This feature makes the K5M more suitable for detecting rapid changes in tear film dynamics. Compared to the RT-7000  provides a single NIBUT reading, the K5M offers two distinct measurements per eye: NIBUTf (the time taken for the first appearance of a break in the tear film) and NIBUTav (the average of the time taken to break-up in all the regions monitored over the duration of the 25 s). In the comparison between K5M and S390L Firefly WDR slitlamp, the K5M is equipped with 22 Placido disk rings^17^. At the same time, the S390L Firefly WDR slitlamp appears to have approximately 23 rings based on the examination images of the patients. The S390L Firefly WDR slitlamp captures images at a frequency of 30 frames per second and has a ring coverage diameter of 8.8 mm under visible light^11^. This feature makes it more similar to the K5M, as both devices are suitable for detecting rapid changes in tear film dynamics. In measuring TMH, the K5M necessitates manual marking of the tear meniscus height. In contrast, the S390L Firefly WDR slitlamp can automatically recognize the tear meniscus shape and obtain measurement data^11,12^. Both instruments effectively provide TMH measurement values.

Both ocular surface analyzers used in our study exhibited good repeatability. In a previous study by Swati et al.^18^it was observed that the IDRA ocular surface analyzer (IDRA-OSA) showed shorter first and average NIBUT compared to the K5M. Additionally, the IDRA exhibited limited reproducibility in TMH measurements. The discrepancies in NIBUT between the IDRA ocular surface analyzer and the K5M could be attributed to variations in algorithm design, sensitivity levels, or differences in subject tolerance to the illumination of the IDRA compared to the infrared emitter source of the K5M, possibly leading to premature tear film break-up^12^. Another potential factor influencing the differing results is the variation in the number of Placido ring bands used in each device, with the K5M utilizing 22 rings compared to roughly eight rings observed in the IDRA based on patient examination images^12^.

Previous studies have suggested that variations in measurement principles, operational procedures, data analysis, and processing methods among different models of ocular surface analysis devices can influence the repeatability of the results^11^. It is acknowledged that the sample size of this study is relatively small. Therefore, further testing and reporting are needed to obtain more comprehensive findings that can be generalized to a larger population. The limited sample size could be considered a limitation of the study. Research by Singh et al.^19^ indicates that the NIBUT measurements by IDRA were lower than those by the K5M. However, their study found that the K5M and IDRA demonstrated acceptable consistency and reliability for NIBUT measurements within the standard group. In contrast, the results were different for the dry eye group. It is important to note that this study did not include the simultaneous assessment of both dry eye patients and non-dry eye patients, which could be considered another limitation.

The findings of this study provide valuable insights for guiding the usage of DED diagnostic tools in complex clinical scenarios. It is vital to employ these instruments cautiously to ensure accurate measurements and reliable data analysis. Nevertheless, these findings are essential in using DED assessment equipment for managing challenging clinical cases. Future investigations could explore different population groups to investigate potential variations in the results. In conclusion, the study demonstrates that S390L Firefly WDR slitlamp exhibits good repeatability and high agreement with the K5M for tear film-related parameter measurements. The S390L Firefly WDR slitlamp can be considered a viable alternative to the K5M in clinical settings.

## Data Availability

Anonymized datasets generated and analyzed during the current study will be made available on reasonable request by the corresponding author (Guanghao Qin, qinguanghao2020@163.com).

## References

[1] Wolffsohn, J. S. et al. TFOS DEWS II diagnostic methodology report. *Ocul Surf.* **15**, 539–574 (2017). 28736342 10.1016/j.jtos.2017.05.001

[2] Kojima, T. et al. A new noninvasive tear stability analysis system for the assessment of dry eyes. *Invest. Ophthalmol. Vis. Sci.* **45**, 1369–1374 (2004). 15111590 10.1167/iovs.03-0712

[3] El Barche, F. Z. et al. Automated tear film break-up time measurement for dry eye diagnosis using deep learning. *Sci. Rep.* **14**, 1–10 (2024). 38778145 10.1038/s41598-024-62636-5 PMC11111799

[4] Kanclerz, P., Bazylczyk, N. & Radomski, S. A. Tear film stability in patients with symptoms of dry eye after instillation of dual polymer hydroxypropyl guar/sodium hyaluronate vs single polymer sodium hyaluronate. *Int. Ophthalmol.* **44**, 1–9 (2024). 38653918 10.1007/s10792-024-03061-5

[5] Willcox, M. D. P. et al. TFOS DEWS II tear film report. *Ocul Surf.* **15**, 366–403 (2017). 28736338 10.1016/j.jtos.2017.03.006 PMC6035753

[6] Zeri, F., Rizzo, G. C., Ponzini, E. & Tavazzi, S. Comparing automated and manual assessments of tear break-up time using different non-invasive devices and a fluorescein procedure. *Sci. Rep.* **14**, 1–13 (2024). 38291100 10.1038/s41598-024-52686-0 PMC10827797

[7] King-Smith, P. E. et al. The thickness of the human precorneal tear film: evidence from reflection spectra. *Invest. Ophthalmol. Vis. Sci.* **41**, 3348–3359 (2000). 11006224

[8] Craig, J. P. et al. TFOS DEWS II definition and classification report. *Ocul Surf.* **15**, 276–283 (2017). 28736335 10.1016/j.jtos.2017.05.008

[9] Sweeney, D. F., Millar, T. J. & Raju, S. R. Tear film stability: a review. *Exp. Eye Res.* **117**, 28–38 (2013). 23973716 10.1016/j.exer.2013.08.010

[10] Vidal-Rohr, M., Wolffsohn, J. S., Davies, L. N. & Cerviño, A. Effect of contact lens surface properties on comfort, tear stability and ocular physiology. *Cont. Lens Anterior Eye*. **41**, 117–121 (2018). 28927731 10.1016/j.clae.2017.09.009

[11] Ballesteros-Sánchez, A., Gargallo-Martínez, B., Gutiérrez-Ortega, R. & Sánchez-González, J. M. Intraobserver repeatability assessment of the S390L firefly WDR slitlamp in patients with dry eye disease: objective, automated, and noninvasive measures. *Eye Cont. Lens* **49**, 283–291 (2023). 10.1097/ICL.0000000000001001 37171516

[12] Chan, Y. Ocular surface parameters repeatability and agreement —a comparison between Keratograph 5M and IDRA. *Cont. Lens Anterior Eye*10.1016/j.clae.2024.102281 (2024). 39097427 10.1016/j.clae.2024.102281

[13] Garcı, V., Talens-estarelles, C. & Cervin, A. Repeatability of non-invasive Keratograph break-up time measurements obtained using oculus Keratograph 5M. **4**, 2473–2483 (2021). 10.1007/s10792-021-01802-4 33728492

[14] Downie, L. E. Automated tear film surface quality breakup time as a novel clinical marker for tear hyperosmolarity in dry eye disease. *Invest. Ophthalmol. Vis. Sci.* **56**, 7260–7268 (2015). 26544794 10.1167/iovs.15-17772

[15] Hong, J. et al. Assessment of tear film stability in dry eye with a newly developed Keratograph. *Cornea* **32**, 716–721 (2013). 23132457 10.1097/ICO.0b013e3182714425

[16] Lee, R. & Yeo, S. Agreement of noninvasive tear break-up time measurement between Tomey RT-7000 auto refractor-keratometer and oculus Keratograph 5M. 1785–1790 (2016). 10.2147/OPTH.S110180 PMC5033501 27695283

[17] Ortiz-Toquero, S., Rodriguez, G., de Juan, V. & Martin, R. Repeatability of placido-based corneal topography in keratoconus. *Optom. Vis. Sci. Off Publ Am. Acad. Optom.* **91**, 1467–1473 (2014). 10.1097/OPX.0000000000000421 25343684

[18] Repeatability reproducibility and agreement between three different diagnostic imaging platforms for tear film evaluation of normal and dry eye disease. 10.1038/s41433-022-02281-2 PMC10333265 36261494

[19] Singh, S. et al. Repeatability of non-invasive tear film evaluation in healthy rabbit eyes repeatability of non-invasive tear film evaluation in healthy rabbit eyes. *Curr. Eye Res.* **48**, 699–703 (2023). 37025013 10.1080/02713683.2023.2200915

